# Impact of Simulated Intestinal Fluids on Dissolution, Solution Chemistry, and Membrane Transport of Amorphous Multidrug Formulations

**DOI:** 10.1021/acs.molpharmaceut.1c00480

**Published:** 2021-10-06

**Authors:** Mira El Sayed, Amjad Alhalaweh, Christel A. S. Bergström

**Affiliations:** † Department of Pharmacy, Biomedical Centre, Uppsala University, P.O. Box 580, Uppsala SE-751 23, Sweden; ‡ Recipharm OT Chemistry AB, Uppsala SE-754 50, Sweden

**Keywords:** multidrug formulations, fixed dose combination, amorphous, FaSSIF, supersaturation, solubility, flux, membrane transport

## Abstract

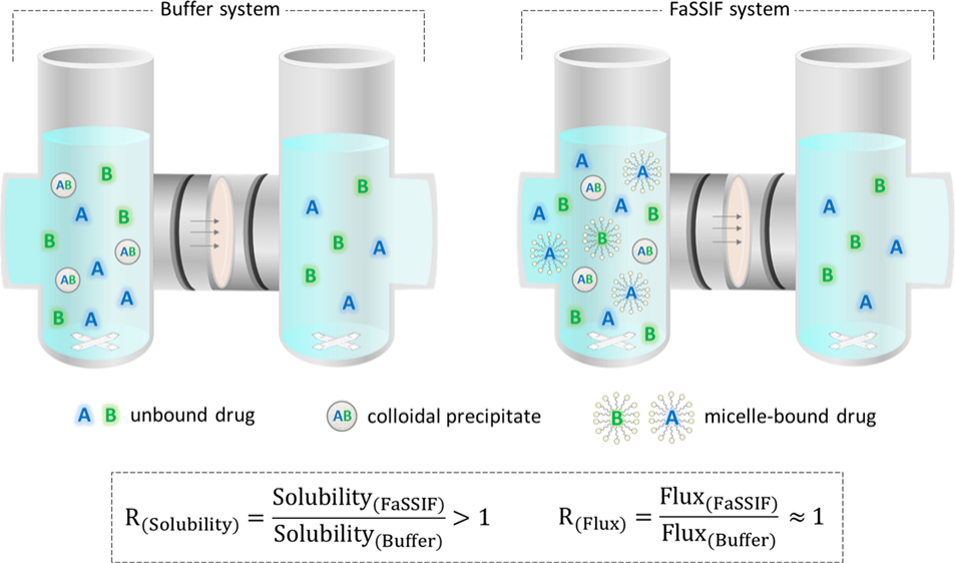

The solution behavior and membrane transport of multidrug formulations were herein investigated in a biorelevant medium simulating fasted conditions. Amorphous multidrug formulations were prepared by the solvent evaporation method. Combinations of atazanavir (ATV) and ritonavir (RTV) and felodipine (FDN) and indapamide (IPM) were prepared and stabilized by a polymer for studying their dissolution (under non-sink conditions) and membrane transport in fasted state simulated intestinal fluid (FaSSIF). The micellar solubilization by FaSSIF enhanced the amorphous solubility of the drugs to different extents. Similar to buffer, the maximum achievable concentration of drugs in combination was reduced in FaSSIF, but the extent of reduction was affected by the degree of FaSSIF solubilization. Dissolution studies of ATV and IPM revealed that the amorphous solubility of these two drugs was not affected by FaSSIF solubilization. In contrast, RTV was significantly affected by FaSSIF solubilization with a 30% reduction in the maximum achievable concentration upon combination to ATV, compared to 50% reduction in buffer. This positive deviation by FaSSIF solubilization was not reflected in the mass transport–time profiles. Interestingly, FDN concentrations remain constant until the amount of IPM added was over 1000 μg/mL. No decrease in the membrane transport of FDN was observed for a 1:1 M ratio of FDN-IPM combination. This study demonstrates the importance of studying amorphous multidrug formulations under physiologically relevant conditions to obtain insights into the performance of these formulations after oral administration.

## Introduction

Fixed dose combinations (FDCs) are routinely used to achieve and improve complex medication regimens and to standardize prescribing practices.^1,2^ Clinical guidelines underpin their use for treating chronic diseases such as tuberculosis, acquired immunodeficiency syndrome, and hypertension.^3−5^ They improve patient compliance to medication and reduce dispensing and supervision costs in health care systems. The World Health Organization classifies many of them as essential medicinal products.^6^

However, formulations of FDCs are not widely investigated in the literature.^7−11^ Only a few studies present the powder dissolution and supersaturation of multidrug formulations,^12−14^ although drugs formulated together may impact the supersaturation of each other. The resulting effects depend on the inherent properties of the drugs such as the extent of ionization and drug miscibility.^12−14^ For instance, the miscible and non-ionizable drugs atazanavir and ritonavir when formulated together reduce the supersaturation of each other compared to formulations containing the individual drugs separately. It has been suggested that this is a result of their mixing in the drug-rich phase, which leads to a decrease in the bulk solution concentrations. In contrast, the presence of an ionizable drug (diclofenac) with ritonavir results in no reduction in the bulk solution concentration of either drug.^13^ Also, it was shown that excipients in the formulation may partition into the colloidal phase and lead to a reduced supersaturation of drugs and thereby membrane transport.^15,16^ Furthermore, the size of the colloidal precipitate may impact the membrane transport of the drug and its bioavailability.^15,17,18^

Amorphous solid dispersions (ASDs) are amorphous blends of drugs molecularly dispersed in a polymeric matrix. The matrix physically stabilizes the amorphous form in the solid state but also stabilizes the supersaturation of the drugs when in solution.^19^ It is well documented that ASDs and salt forms can generate supersaturating solutions of the drugs.^20,21^ The supersaturation reaches a maximum when the liquid–liquid phase separation (LLPS) occurs, resulting in a drug-poor phase (aqueous bulk) and a drug-rich one (amorphous colloidal aggregates).^22^ The selection of the polymer is critical, as it may reduce the maximum achievable concentration of the drug if it partitions into the drug-rich phase.^23,24^ Supersaturated formulations have a higher membrane transport than the crystalline-based ones because the supersaturated state has a higher thermodynamic activity than the saturated state.^16,25,26^ Indeed, the increased amount of the dissolved drug due to the solubilization by biological fluid components or solubilizing agents does not increase the membrane transport rate of the drug.^27,28^ Also, the free drug available for absorption from supersaturated solutions reaches a maximum when the amorphous solubility is attained; this applies whether the drug is formulated either alone or in combination with another drug.^12^

Hence, there is a need to make use of the ASD technology and advance the knowledge of amorphous multidrug formulations as a means to formulate drugs with poor solubility. In contrast to multidrug formulations, the impact of simulated intestinal fluids on single drug formulation is extensively covered in the literature.^29−32^ Biorelevant media improve the solubility of drugs in comparison to plain buffer solutions, but the degree of improvement is dependent on the inherent properties of the drugs. Neutral or positively charged drugs are presumed to have a high increase in solubility due to more efficient solubilization.^29^ The biorelevant media components also affect the solubility and crystallization kinetics of the drugs. For example, it was reported that the content of lecithin and taurocholate in simulated intestinal fluids affects the amount of drug dissolved and the induction time of crystallization.^33^

Thus, conducting studies on the supersaturation of multidrug formulations in biorelevant media is essential to investigate their performance in a medium mimicking the intestinal conditions. Taylor and colleagues have demonstrated that drug transport across a membrane is not affected by the media composition nor by the solubilizing additives present in solution.^27,28^ Rather, it is the solid form of the drug in equilibrium or metastable equilibrium with the bulk aqueous phase that has the greatest impact on drug transport across a membrane. It was also found that the membrane transport of the amorphous form of a drug (atazanavir or posaconazole) is the same in buffer and FaSSIF and always higher than that of their crystalline form.^27^ In another example, amorphous multidrug formulations of atazanavir and ritonavir exhibit a decrease in membrane transport in buffer. The decrease is proportional to the molar ratio of the drugs present in the formulation, where ideal mixing between the two drugs in the drug-rich phase occurs.^12^

In light of current needs, our study had four aims. These were: (i) to study the dissolution, amorphous solubility and supersaturation of multidrug formulations in simulated intestinal fluid and contrast this behavior to that in buffer; (ii) to investigate the impact of amorphization and biorelevant media on the supersaturation and solution behavior of the individual model drugs compared to multidrug formulations; (iii) to establish a thermodynamic model that predicts the performance of multidrug formulations in biorelevant media; and (iv) to understand the impact of solution media and drug combination on the mass transport (flux) of the drugs across a membrane. Two prescription drug combination models were selected for this study. The first model combined atazanavir (ATV) and ritonavir (RTV), an antiretroviral therapy, in which low-dose RTV is co-administered with ATV to optimize its pharmacokinetic parameters and efficacy.^34^ The second model combined felodipine (FDN) and indapamide (IPM), a widely prescribed antihypertensive combination previously studied in our group.^14^ ATV and RTV are two drugs known to decrease the maximum achievable concentration of each other when combined.^12^ In contrast, FDN and IPM in combination show different degrees of solubility decrease from the supersaturated solutions, with a pronounced and statistically significant decrease for IPM but not for FDN being observed.^14^

## Experimental Section

### Materials

**Figure 1:**
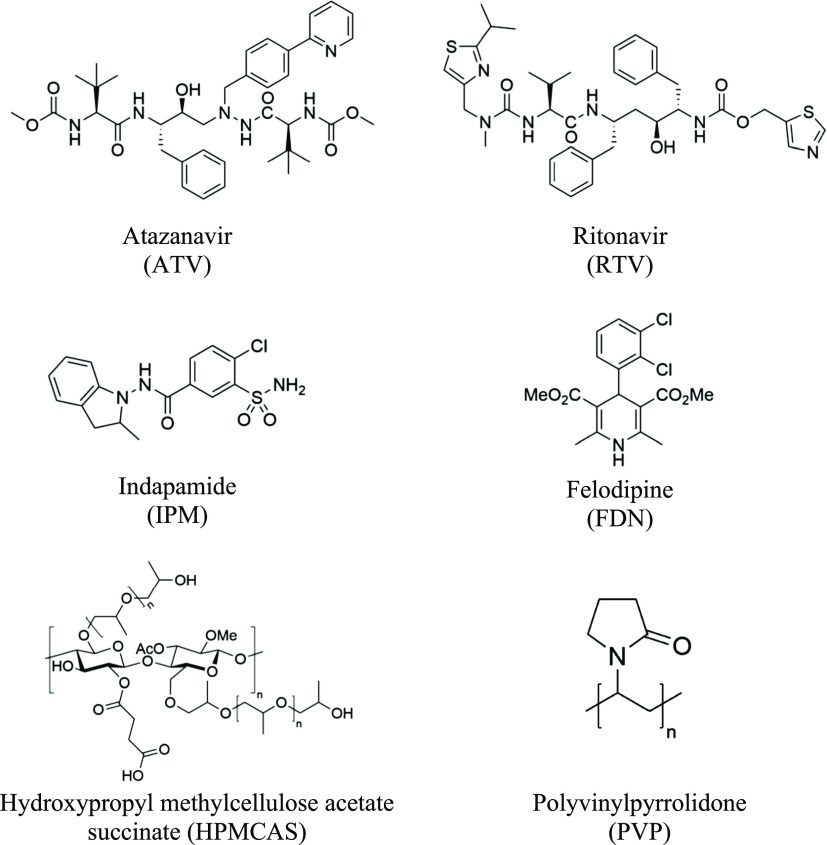
Chemical structures of the model drugs and polymers in this study.

RTV and ATV sulfate were purchased from Attix Pharmaceuticals (Toronto, Canada) and Chemtronica (Stockholm, Sweden). IPM was obtained from Recipharm (Milan, Italy). FDN was a gift from AstraZeneca (Mölndal, Sweden). Hydroxypropyl methylcellulose acetate succinate (HPMCAS: Shin-Etsu AQOAT, Type AS-MF) polymer was a gift from Shin-Etsu Chemical Co. (Tokyo, Japan). Polyvinylpyrrolidone (PVP: Kollidon, Type 17-PF) polymer was a gift from BASF Ltd. (Stockholm, Sweden). The chemical structures of the model drugs and polymers are presented in Figure 1. Acetonitrile, methanol, and dichloromethane were acquired from CARLO ERBA Reagents S.A.S. (Barcelona, Spain) or Sigma-Aldrich (Stockholm, Sweden). Sodium hydroxide pellets, sodium chloride, and sodium dihydrogen phosphate dihydrate were purchased from CARLO ERBA Reagents S.A.S. (Barcelona, Spain), Sigma-Aldrich (Stockholm, Sweden), and Merck (Darmstadt, Germany), respectively. FaSSIF-V1 powder was procured from biorelevant.com (Croydon, UK). Spectra/Por 1 regenerated cellulose membrane with a molecular weight cutoff value of 6–8 kD was purchased from VWR (Stockholm, Sweden). Milli-Q water was used for all aqueous solutions. All drugs were received and used as received except for ATV. The amorphous base form of ATV was prepared as described earlier;^12^ briefly, the drug was dissolved in methanol, then the solution was titrated with 0.1 M sodium hydroxide until the amorphous base precipitated. The crystalline form was then obtained by adding a water/methanol (1:1 v/v) mixture to the amorphous powder while stirring for 96 h at room temperature. Molecular properties of the model drugs were calculated with the software ADMET predictor (SimulationPlus, CA) using molecules as the structure data file.

## Methods

### Media Preparation

Media in the dissolution and flux experiments were either 50 mM pH 6.5 phosphate buffer or FaSSIF. The aqueous buffer was prepared using sodium hydroxide pellets, sodium chloride, and sodium dihydrogen phosphate dihydrate. FaSSIF medium was prepared by directly dissolving the FaSSIF-V1 powder into the previously stated buffer components as per the instructions of the manufacturer. The version of FaSSIF used in this study contains 3 mM sodium taurocholate and 0.75 mM lecithin.

### High-Performance Liquid Chromatography

The concentration of the model drugs was determined using an Agilent 1290 high-performance liquid chromatography (HPLC) system (Agilent Technologies, Santa Clara, CA) equipped with a DAD detector and Zorbax Column Eclipse XDB-C18 (4.6 mm × 15 or 25 cm), 5-μm particle size. Injection volume was set to 20 μL. The column temperature was maintained at 30 °C and the run time was 15 min. Conditions for the analysis of the drugs are described in Table 1. Calibration curves covering the concentration range of the drugs in the supernatant were constructed and exhibited good linearity (*R*^2^ ≥ 0.999). If required, drug solutions were diluted with the mobile phase to obtain concentrations within the limits of the calibration curves.

**Table 1:** Chromatographic Conditions for Determining the Concentration of the Model Drugs by HPLC

	drug combination	
parameter	ATV-RTV	FDN-IPM
flow rate (mL/min)	0.75	1
UV wavelength (nm)^a^	240	250
mobile phase (%v/v)^b^	45:55	40:60

a 214 nm was used for samples from flux experiments of ATV, RTV, and FDN.

b Water/acetonitrile.

### Differential Scanning Calorimetry

Differential scanning calorimetry (DSC) measurements were carried out using a TA Instruments Q2000 equipped with a refrigerated cooling system. The chamber was purged with nitrogen at a flow rate of 50 mL/min during the testing. The system was calibrated for temperature and enthalpy using indium and for heat capacity using sapphire. Thermodynamic parameters were calculated using TA Universal Analysis 2000 software. Samples (1–5 mg) were weighed into non-hermetic aluminum pans and an empty pan was used for reference. The onset of melting (*T*_m_) and heat of fusion (Δ*H*_f_) were determined using a heating rate of 10 °C/min. A heat–cool–heat cycle was performed to determine the glass transition temperature (*T*_g_). The crystalline form of the drugs was heated to a temperature equal to 2 °C above their melting onset, where the system was left at the isothermal step for 2 min. The samples were then cooled to −20 °C at a ramp rate of 20 °C/min. *T*_g_ values were recorded as the step-change inflection point from second heating scans at a heating rate of 20 °C/min, instantly after cooling. The prepared solid dispersions were characterized by cooling the samples to −20 °C after which the samples were heated at 20 °C/min to well above the peak melting temperature of the drugs.

### Powder X-ray Diffraction

The powder X-ray diffraction (PXRD) diffractograms of ATV and RTV were recorded using a Twin–Twin diffractometer (Bruker, Coventry, UK) equipped with a sample rotator. The solid samples were placed on silicon sample holders during the measurement. The tube voltage and amperage were set to 40 kV and 40 mA, respectively, and the emitted Cu Kα radiation was 1.54 Å. Diffraction images were collected with a 2θ scan range from 5 to 40° at a scanning step of 0.02° and 0.3 s exposure time at each step. Primary and secondary divergence slits of 0.40 and 2.48 mm, respectively, were used.

### Preparation of ASDs

ASDs of single- and two-drug formulations were prepared by rotary evaporation. For ATV and RTV, the solutions were made by dissolving a total of 1 g of the mixture containing 90% w/w PVP and 10% w/w of either ATV, RTV, or a mixture of both at various molar ratios (25:75, 50:50, and 75:25), in 5 mL of methanol. The solvent was removed using a rotary evaporator R-210 (BÜCHI Labortechnik AG, Switzerland) at 40 °C and the resulting powder was placed under vacuum for 24 h to remove any residual solvent. For FDN and IPM, formulations containing 80% w/w HPMCAS and 20% w/w of either FDN, IPM, or a mixture of both at various molar ratios (25:75, 50:50, 75:25, and 90:10) were prepared. The polymer and drug(s) were first dissolved in amethanol/dichloromethane (1:1 v/v) solution and then the solvent was removed as described above. Table 2 lists the ASDs and their compositions. These compositions were used in our previous work and were prepared for this study to compare their solution behavior in FaSSIF to what was observed earlier in buffer.^12,14^ The solids were then collected, sealed with parafilm, and stored at −20 °C. All solids were analyzed by DSC for solid-state characterization.

**Table 2:** Formulation of the ASDs with the Weight Ratio of Each Component

polymer-drug system	polymer (% w/w)	drug(s) (% w/w)	drug ratio^a^
PVP-ATV	90	10	n/a
PVP-RTV	90	10	n/a
PVP-ATV-RTV	90	10	25:75
PVP-ATV-RTV	90	10	50:50
PVP-ATV-RTV	90	10	75:25
HPMCAS-FDN	80	20	n/a
HPMCAS-IPM	80	20	n/a
HPMCAS-FDN-IPM	80	20	25:75
HPMCAS-FDN-IPM	80	20	50:50
HPMCAS-FDN-IPM	80	20	75:25
HPMCAS-FDN-IPM	80	20	90:10

a Only applicable for formulations containing two drugs. n/a: not applicable.

### Crystalline Solubility Determinations

Crystalline solubility was measured by adding excess solids (10–20 mg) to 5 mL or 15 mL of either 50 mM pH 6.5 phosphate buffer or FaSSIF media. Samples were placed in an oven at 37 °C and stirred continuously for 48 h for the buffer and 24 h for FaSSIF. The supernatant was separated from the excess solid for ATV and RTV (15 mL each) by ultracentrifugation at 217,290*g* for 30 min in an Optima L-60 (Beckman Coulter, Inc., Brea, CA) equipped with Swinging-Bucket Rotor SW41 Ti. FDN and IPM (5 mL each) were ultracentrifuged at 193,911*g* in an SW55 Ti rotor. The concentration of the supernatant was thereafter determined by HPLC as described above. The solid forms at the end of the experiment were analyzed by DSC.

### Amorphous Solubility Determinations

The amorphous solubility of the drugs was determined in both 50 mM pH 6.5 phosphate buffer and FaSSIF media. Stock solutions with different concentrations of the model drugs were prepared in methanol. The concentrations were varied to ensure that amorphous solubility of the drug was exceeded and the methanol content was maintained to be not ≥1% v/v. The appropriate amount from the methanol stock was introduced to 5 mL or 15 mL of the desired aqueous medium at 37 °C while stirring until turbidity. FDN and IPM experiments were performed in the presence of 25 μg/mL of HPMCAS pre-dissolved in the aqueous media. The supernatant was then separated by ultracentrifugation and analyzed by HPLC as described above.

### Dissolution of Formulations

Powder dissolution experiments of the ASDs were performed under non-sink conditions at 37 °C. Around 80–90 mg of powder was added to 4 mL of FaSSIF media, and samples were stirred at 300 rpm on a Variomag multipoint magnetic stirrer. The amount of ASD added was selected so that the theoretical concentrations of both components in the formulation would be above the reported amorphous solubility. Three independent experiments were performed at each time point (5, 30, 60, and 120 min). Thereafter, the samples were ultracentrifuged as described above; the supernatant layer was diluted with the mobile phase if necessary and analyzed by HPLC.

### Drug Transport Experiments

The diffusive flux, *J*, at amorphous solubility for both drug combinations, was studied in buffer and FaSSIF. Experiments were conducted in a MicroFLUX setup (Pion Inc., USA). The donor and receiver compartments were separated by a regenerated cellulose membrane (MWCO 6–8 kDa); the surface area of the membrane available for mass transport was 1.54 cm^2^. Membranes were pre-soaked in water for 3 h. The donor and receiver compartments were agitated by magnetic stirring at 300 rpm. Each compartment contained 16 mL of media (either buffer or FaSSIF) and maintained at 37 °C. To prevent crystallization, HPMCAS was added to both compartments: 100 μg/mL for ATV and RTV and 25 μg/mL for FDN and IPM. The concentrated stock solutions of the drugs were prepared in methanol and then added into the donor side to achieve concentrations 20% above the amorphous solubility of eachdrug for the drug alone experiments; the amount of methanol in the solution was kept below 1%. In the combination experiments, a mass balance was applied in preparing the mixed stock solutions to achieve a 50:50 weight ratio of drug precipitates, maintaining the methanol amount below 1% as well. Drug permeation to the receiver side was monitored by withdrawing 100 μL aliquots at different time points and the concentration was determined by HPLC. The concentration was then plotted as a function of time. The slope of each plot was converted to diffusive flux (*J*) by factoring in the volume of the media and cross-sectional area of the membrane.

### Statistical Analysis

All results are expressed as mean values with a standard deviation in parentheses. Unpaired *t*-tests (two-tailed) were used at a 95% confidence interval to evaluate the differences between the samples in buffer and FaSSIF, or from single- and two-drug formulations, during dissolution and flux experiments. Values of *p* < 0.05 were considered statistically significant. Pearson’s correlation test was applied to determine the linear association between the ratio of the drug content in the formulation and the maximum achievable concentration of the drug. The analysis was conducted using GraphPad Prism software (version 9.0.0).

## Results

### Characterization of Model Drugs and Formulations

The four drugs have different physiochemical properties. All are neutral at pH 6.5 of the study buffer and FaSSIF medium. Thermal properties of ATV and RTV were measured by DSC (Supporting Information, Figures S1 and S2). The two drugs were in the crystalline form, which was also confirmed by PXRD. *T*_m_, *T*_g_, and Δ*H*_f_ values were in agreement with previously reported ones (Table 3).^12,20,35^ Despite similarities in their ionization and chemical structures, ATV and RTV had significantly different *T*_m_ and Δ*H*_f_ values: ATV had a *T*_m_ of 483 K and Δ*H*_f_ of 52.8 kJ·mol^–1^, whereas RTV had both lower *T*_m_ (399 K) and Δ*H*_f_ (49.7 kJ·mol^–1^). The resulting formulations containing ATV and/or RTV were amorphous as no melting endotherm was observed by DSC (Supporting Information, Figure S3).^12^ Detailed characterization of the crystalline FDN and IPM drugs and their corresponding amorphous formulations are presented in our previous work.^14^

**Table 3:** Physicochemical Properties of the Model Drugs^a^

drug	pharmacological class	ionization	p*K*_a_	MW (g·mol^–1^)	log P	Δ*H*_f_ (kJ·mol–^1^)	*T*_m_ (K)	*T*_g_ (K)
ATV	anti-HIV	weak base	4.4	704.86	3.4	74 (1.9)	484 (0.6)	377
RTV	anti-HIV	weak base	3.7	720.95	4.2	49.7 (7.5)	399 (0.2)	322
FDN	CCB	neutral	n/a	384.26	3.9	29.2 (8.2)	420 (0.1)	318
IPM	TLD	weak acid	8.8	365.84	2.2	32.3 (0.4)	435 (0.4)	373

a HIV: human immunodeficiency virus, CCB: calcium channel blocker, TLD: thiazide-like diuretic, p*K*_a_: acid dissociation constant, MW: molecular weight, log *P*: log partition coefficient between octanol and water, Δ*H*_f_: heat of fusion, *T*_m_: onset of melting, *T*_g_: glass transition temperature at inflection and n/a: not applicable. Standard deviation reported based on triplicates in parenthesis. Details of FDN and IPM characterization can be found in our previous work.^14^

### Solubility of Crystalline and Amorphous Forms of the Drugs

Solubilities of the crystalline and amorphous forms were determined in buffer and FaSSIF (Table 4). In both media, amorphization clearly increased the solubility of the four drugs compared to their crystalline counterparts, this was evident by the *S*_A_/*S*_C_, the ratio of amorphous to crystalline solubility (Table 4). The solubilization advantage contributed by FaSSIF is designated as *R*_(Solubility)_ in Table 4. The solubilization of FDN in FaSSIF was the highest—68 and 19 times higher in FaSSIF as compared to buffer for the crystalline and amorphous forms, respectively. The drugs that were efficiently solubilized by FaSSIF (FDN and RTV) were less solubilized in the amorphous form than in their crystalline counterpart.

**Table 4:** Crystalline and Amorphous Solubility in 50 mM pH 6.5 Phosphate Buffer and FaSSIF^a^

	crystalline solubility (μg/mL)		amorphous solubility (μg/mL)		*R*(solubility)^b^		*S*_A_/*S*_C_^c^	
drug	buffer	FaSSIF	buffer	FaSSIF	crystalline solubility	amorphous solubility	buffer	FaSSIF
ATV	1.1 (0.4)	1.5 (0.1)	80 (0.6)	113 (1.8)	1.4	1.4	72.7	75.3
RTV	2.2 (0.5)	5.8 (0.2)	29 (0.1)	51 (1.2)	2.6	1.8	13.2	8.8
FDN	0.6 (0.1)	41 (1.1)	7 (0.1)	154 (1)	68.3	18.9	11.7	3.2
IPM	114 (2.2)	117 (0.2)	1087 (29.8)	1182 (26)	1.0	1.1	9.5	10.1

a Amorphous solubility was determined by the antisolvent method. The results of the crystalline solubility and amorphous solubility of FDN and IPM determined in 50 mM pH 6.5 phosphate buffer are reported from our previous work.^14^ Standard deviation reported based on triplicates in parenthesis.

b *R*_(solubility)_ = solubility_(FaSSIF)_/solubility_(buffer)_.

c *S*_A_/*S*_C_: ratio of amorphous to crystalline solubility.

### Dissolution of ATV-RTV in FaSSIF

**Figure 2:**
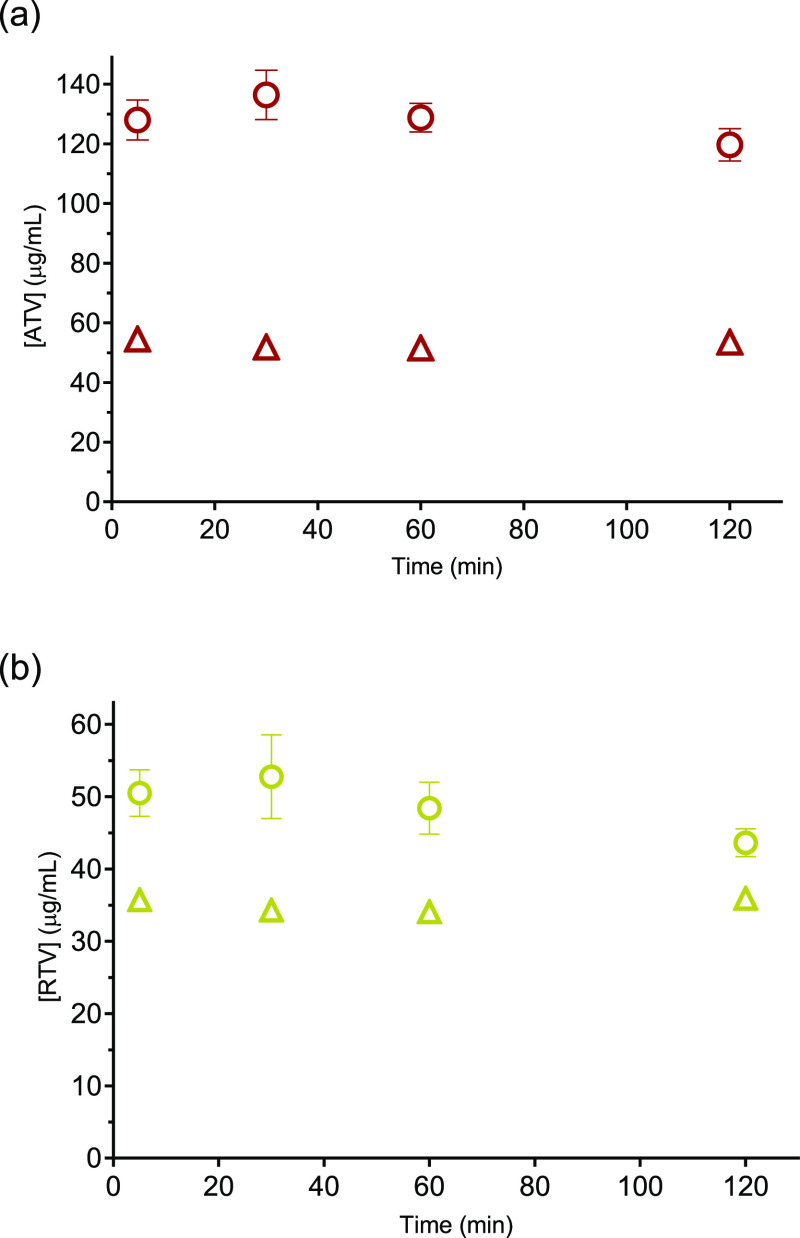
Dissolution profiles of amorphous formulations of ATV and RTV. Formulations containing 90% PVP and 10% drug(s) in FaSSIF for (a) ATV in red and (b) RTV in yellow. Circles (◯) represent drug alone (ATV or RTV) and triangles (△) represent the 1:1 M ratio of ATV and RTV. Error bars (three replicates) show standard deviations (error bars are smaller than the symbols for some measurements).

Next, we investigated the impact of the second component on the maximum achievable supersaturation of the first component by looking at the powder dissolution of ATV alone (PVP-ATV), RTV alone (PVP-RTV), and a 1:1 M ratio of ATV and RTV (PVP-ATV-RTV). Figure 2 shows the dissolution profiles in FaSSIF under non-sink conditions of single and combination drug formulations over a period of 120 min. ATV in PVP-ATV attained a value of 119 μg/mL and RTV in PVP-RTV reached a steady state at 53 μg/mL with no further increase in the free drug concentration. These results agree with the amorphous solubility values measured by the antisolvent method for each component in FaSSIF. On the other hand, the supersaturation from the formulation containing a 1:1 M ratio of ATV and RTV was lower than the supersaturation from the single drug formulations. The maximum achievable concentration of ATV and RTV decreased by 50 and 30%, respectively, of the corresponding concentrations achieved by the single drug formulations under the same conditions (Figure 2).

### Dissolution of FDN-IPM in FaSSIF

**Figure 3:**
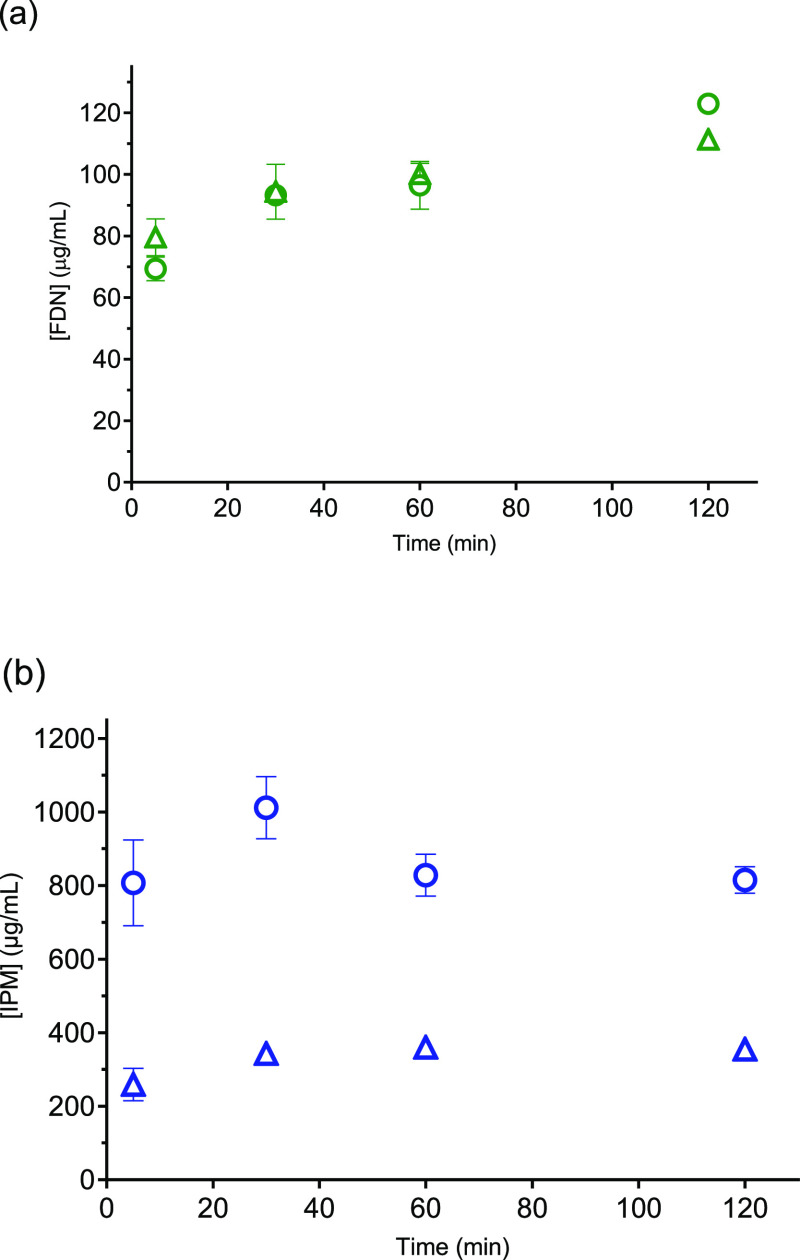
Dissolution profiles of amorphous formulations of (a) FDN (green) and (b) IPM (blue). Formulations contained 80% HPMCAS and 20% drug(s) in FaSSIF. Circles (◯) represent FDN or IPM alone and triangles (△) a 1:1 M ratio of FDN and IPM. Error bars (three replicates) show standard deviations (error bars are smaller than the symbols for some measurements).

Dissolution profiles of FDN alone (HPMCAS-FDN), IPM alone (HPMCAS-IPM), and 1:1 M ratio of FDN and IPM (HPMCAS-FDN-IPM) in FaSSIF are shown in Figure 3. A slight reduction in the supersaturation of FDN was observed when combined with IPM at 120 min. The IPM concentration from the formulation containing IPM alone attained a solubility value of 815 μg/mL in FaSSIF after 120 min. In contrast, the IPM concentration from the 1:1 M ratio of FDN-IPM reached a steady state at a solubility value of 354 μg/mL.

### Flux in Response to Supersaturation and Solubilization

**Figure 4:**
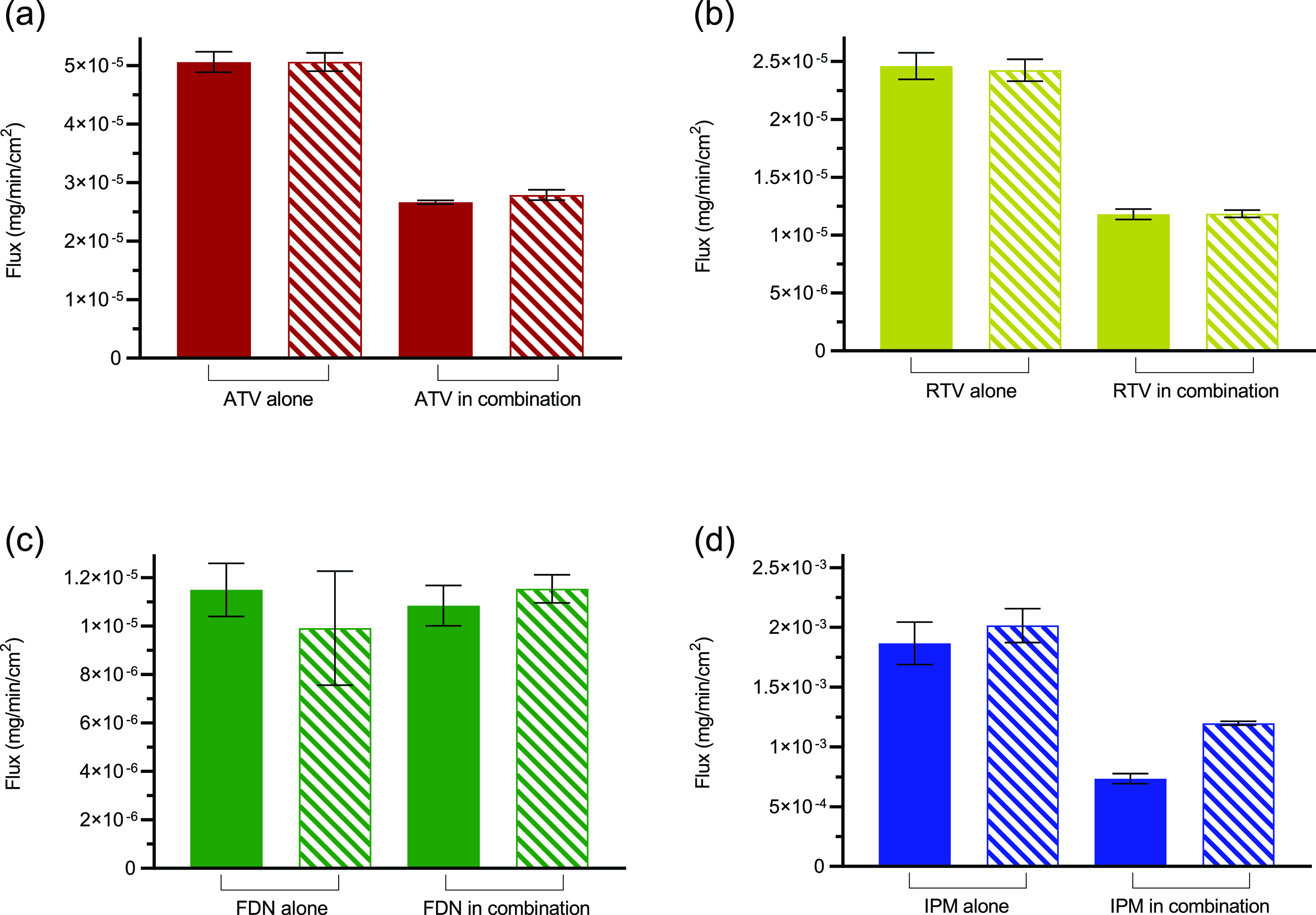
Flux measurements of ATV, RTV, FDN, and IPM, alone and in combination in two media. Solid bars represent buffer and striped bars, FaSSIF. (a) ATV (red), (b) RTV (yellow), (c) FDN (green), and (d) IPM (blue). Error bars (4 replicates) show standard deviations.

The membrane transport of ATV, RTV, FDN, and IPM—alone and in combination—was studied in buffer and FaSSIF. Whether the drugs were alone or in combination, similarity in transport across the membrane was observed for the two media (Supporting Information, Figure S4). The linear regression analysis and the normalized flux values of the plots are presented in Supporting Information, Tables S1 and S2, respectively. The membrane transport of ATV, RTV, and IPM decreased for the combination formulations studied, whereas the same flux was obtained in both conditions of FDN alone and in combination (Figure 4).

## Discussion

### Solution Performance of Multidrug Formulations

Dissolution studies under non-sink conditions using ASD of multidrug formulations have shown that drugs formulated together with the polymer can precipitate as a colloid in a single phase. This in turn reduces the extent of supersaturation as a function of drug ratio in the formulation.^12,13^ In these studies, it was possible to predict the precipitation of one phase with a simple model that makes use of amorphous solubility and drug ratio in the formulation of the respective drug. This colloid formation is dependent on the ionization properties of the drugs in solution, and their miscibility in the drug-rich phase.^13,14,36^ The maximum achievable concentration of a drug decreases because of adecrease in its chemical potential due to the partitioning of the second component in the drug-rich phase of the first component. Polymers or other components in solution can incorporate in the drug-rich phase thereby reducing the maximum achievable concentration.^15,23,24^ The drug becomes diluted in the drug-rich phase, which determines the drug chemical potential. In fact, the co-existence of water, polymer, and drugs in the drug-rich phase forms a complex system that cannot be related to the solid-state mixture of amorphous multidrug formulation.^37,38^

In light of the complex process of dissolution of amorphous multidrug formulations, it is crucial to investigate the dissolution of multidrug formulations in biorelevant media that incorporate additional colloidal structures from bile.^32^ These media may enhance drug amorphous solubility by micellar solubilization.^33^ Indeed, dissolution in simulated intestinal fluid is affected for instance by whether the drug is administered before or after food intake.^32^ The amount of lipids and bile salts affect the solubility, supersaturation, and crystallization tendency of the drugs from the supersaturated solution.^29,31,33^ This knowledge of the performance of drugs in gastrointestinal fluid is of great relevance to the design of successful multidrug formulations.

### Impact of Amorphization and FaSSIF on Solubilization

The solid-state properties and hydrophobicity of a drug compound are the major factors affecting its solubility.^39^ We therefore investigated the impact of amorphization and solubilization by FaSSIF components on the different drugs. The crystal and solvation contributions to the free energy of the solubilization can be demonstrated using the following equation (eq 1)^40,41^
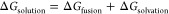
1

Solubilization methods based on physical transformation such as amorphization, eliminate the limitation imposed by the ideal solubility, reducing the barriers to only the solvation contribution (log γ) that can be calculated from this relation (eq 2)^40,41^
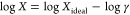
2where *X* is the measured crystalline solubility, γ is the activity coefficient, and *X*_ideal_ is the ideal solubility, quantitatively determined as follows (eq 3)^40,41^
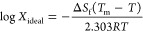
3where Δ*S*_f_, *R*, *T*_m_, and *T* are the melting entropy, the ideal gas constant, the onset of melting, and the experimental temperature (37 °C), respectively.

**Figure 5:**
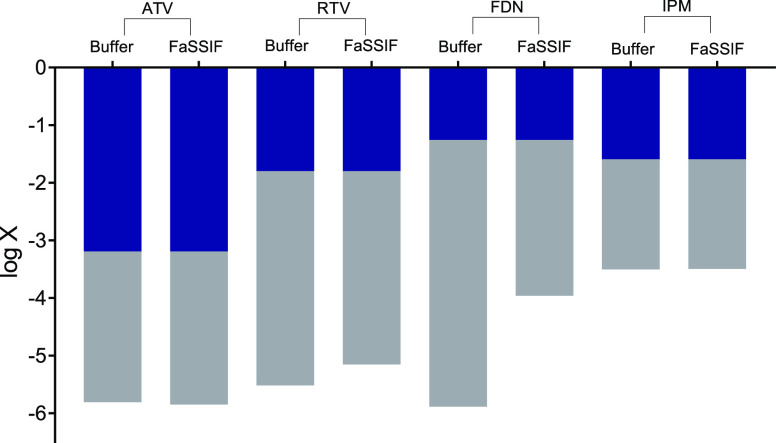
Solubilization barriers to the crystalline solubility of ATV, RTV, FDN, and IPM in buffer and FaSSIF. The bars show the two independent factors that determine solubility, the ideal solubility: log *X*_(ideal)_ (blue), and the activity coefficient: log γ (gray).

**Figure 6:**
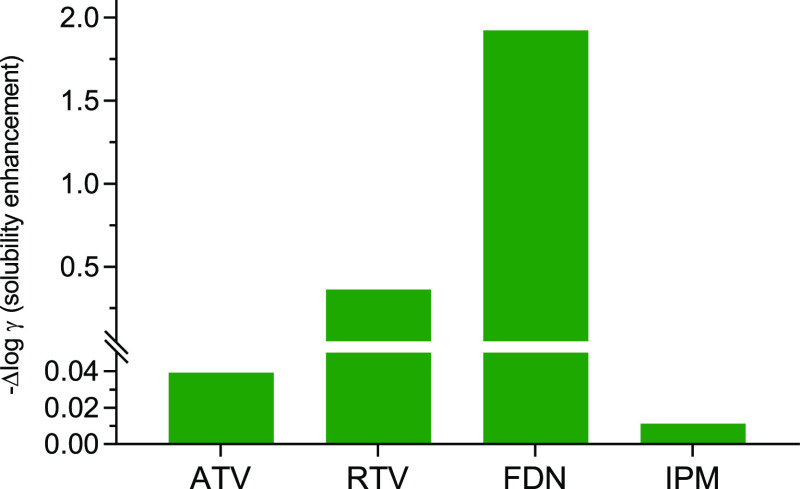
Solubility enhancement by FaSSIF on the solubility of crystalline ATV, RTV, FDN, and IPM.

Figure 5 displays the solubilization barriers induced by the crystallinity and the hydrophobicity of the studied drugs. The calculation of log γ was obtained by the difference between log *X*_(ideal)_ and log *X* as per eq 2. The experimentally determined thermal properties necessary to obtain log *X*_(ideal)_ are listed in Table 3. Calculations of the two solubilization barriers, log *X*_(ideal)_ and log γ, are presented in the Supporting Information, Table S3. ATV has the highest crystal packing contribution; this was reflected in its high *T*_m_ (484 K) and Δ*H*_f_ (74 kJ·mol^–1^) values. This is also clearly seen by the ∼72–75-fold increase in amorphous solubility compared to its crystalline counterpart in both buffer and FaSSIF. FDN has the lowest crystallinity and highest activity coefficients, reflected by a *T*_m_ of 422 K and a Δ*H*_f_ of 29.2 kJ·mol^–1^. Those findings were in agreement with the results presented in Tables 3 and 4. Figure 6 shows the solubility enhancement by FaSSIF on the crystalline solubility of the drugs, calculated by the difference between log γ_(buffer)_ and log γ_(FaSSIF)_. The improved solubility of the compounds by FaSSIF was explained by the formation of micelles composed of sodium taurocholate (a bile salt) and lecithin (a phospholipid). The variations in the composition of the biorelevant medium can lead to differences in the solubility of drugs.^33^

### Solution Performance of the ATV-RTV Combination

**Figure 7:**
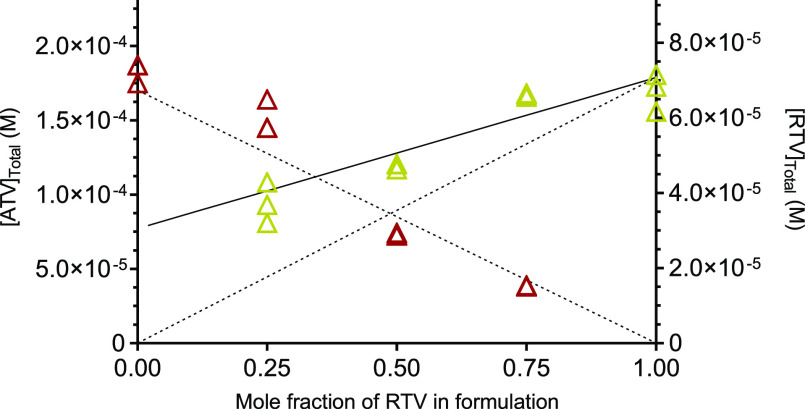
Concentration of ATV (red) and RTV (yellow) in the supernatant layer of ASDs containing both drugs following the dissolution at different molar ratios in FaSSIF. The lines represent the predicted concentrations based on eq 4 (dotted) or eq 5 (solid).

**Figure 8:**
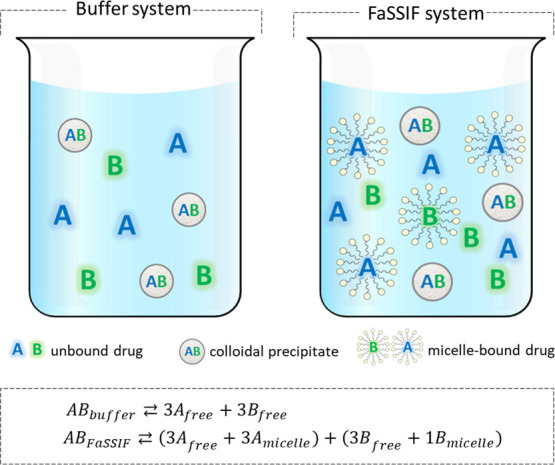
Solution behavior of an ASD containing two drugs, A and B, in buffer and in FaSSIF, assuming both drugs are miscible and neutral at the solution pH. The amount of the molecularly dissolved drug in the aqueous media exceeds the amorphous solubility of both drugs, leading to phase separation and precipitation of AB (as a colloidal precipitate). Drugs A and B are solubilized at different levels by FaSSIF leading to a higher solubility increase of drug A compared to drug B in FaSSIF.

Non-ionized ATV with RTV form a miscible system in their amorphous forms and show a reduction in their maximum achievable concentrations from their combination formulation compared to their single drug formulations.^12^ This decrease in the concentration is proportionally related to the mole fraction of the drugs in the formulations, where (eq 4) predicts the concentrations reached when the drugs were given in combination^12,13^
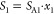
4where, *S*_1_ is the solubility of component 1 in the bulk phase in the presence of a second component in the drug-rich phase; *S*_A1_ is the amorphous solubility of component 1 alone in the study medium; and *x*_1_ is the mole fraction of component 1 present in the mixture. For ATV and RTV, we plotted the concentration of both drugs in FaSSIF against the mole fraction of RTV in formulations of various ratios of ATV and RTV. The maximum achievable concentrations of ATV were well predicted by eq 4 using *S*_A1_ as the amorphous solubility of ATV in FaSSIF (Figure 7). However, the concentrations of RTV were not possible to fit to the measured concentrations using eq 4. The dissolution experiment of the PVP-ATV-RTV system in FaSSIF only reduced the concentration of RTV to 30% as compared to 50% reduction in buffer. This positive deviation is likely due to the efficient solubilization by FaSSIF components, as discussed below in Figure 8. In contrast, the dissolution of ATV in the combination formulation was not affected by FaSSIF, in agreement with the literature.^33^

The relation between the maximum achievable concentration of RTV and its mole fraction in the formulation was evaluated using the Pearson’s correlation test and was demonstrated to be linear (*r* = 0.93). Thus, the extent of micellar solubilization was calculated by the difference in the measured amorphous solubility of RTV from ATV-RTV formulations with varying ATV-RTV ratios in buffer and in FaSSIF assuming the micelle-bound RTV is not changing. Accordingly, the following model (eq 5) was implemented to describe the solubility of RTV which was highly solubilized by FaSSIF to include the micellar contribution (*b*)^42^
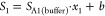
5

Equation 5 was used to estimate the solubility of RTV in the ATV-RTV formulations using the amorphous solubility in buffer (*S*_A1(buffer)_) and the micellar contribution (*b*), which was calculated by the difference in the measured amorphous solubility of RTV in buffer and in FaSSIF (*b* = 3.05 × 10^–5^ M). A better prediction of the solubility of RTV was obtained from eq 5 than eq 4 (Figure 7). Equation 5 might be extended to other surfactant-based solubilization systems in an attempt to predict the maximum achievable concentration of drugs from multidrug formulations that have the same solution behavior of ATV-RTV. The solubility of a particular drug that is solubilized by other biorelevant media or surfactant-based systems can be measured and the micellar contribution (*b*) can be determined in order to predict the maximum achievable concentration of the drug. Depending on the solubility of the drug in FaSSIF, either eq 4 or eq 5 should be used to predict the maximum achievable concentration of the drugs evolving from multidrug formulations with solution behavior similar to that of ATV-RTV combination. This approach could help in the design of amorphous formulations of multicomponent systems; it is a time-saving, material-sparing, and an efficient tool in assessing the solubility of drugs under ASD development.

A schematic illustration of the FaSSIF solubilization effect on drugs with a scenario similar to ATV-RTV from a multidrug formulation is presented in Figure 8 to clarify the solution performance in FaSSIF for this model of multidrug formulation. Molecules of an ASD containing two drugs (A and B) are dissolved in buffer or FaSSIF. In the aqueous buffer, both drugs A and B dissolve as free drugs in the bulk solution and the amount of the drug above the maximum achievable concentration precipitates as colloidal particles that contain both drugs at a defined ratio (assuming that they are miscible in the drug-rich phase). In FaSSIF, the drugs may be solubilized to a different extent. Drug A, herein representing RTV, is more solubilized by FaSSIF compared to drug B, which is reflected as a higher measured concentration of drug A compared to drug B. This clearly shows that FaSSIF exerts a differential solubilization on the two drugs.

### Solution Performance of the FDN-IPM Combination

The maximum achievable concentration of the drugs from the dissolution of the FDN-IPM formulation revealed a slight decrease in the concentration of FDN after 120 min and a 50% decrease in the concentration of IPM compared to the concentrations achieved from the drug-alone formulations. This behavior was also observed in the buffer solution for this combination. The amorphous solubility of IPM and FDN in formulations with a high content of HPMCAS was lower than that measured by the antisolvent method with only 25 μg/mL polymer. It has previously been observed that a high content of HPMCAS reduces the amorphous solubility of the drug due to the partitioning of the polymer in the drug-rich phase.^24^

**Figure 9:**
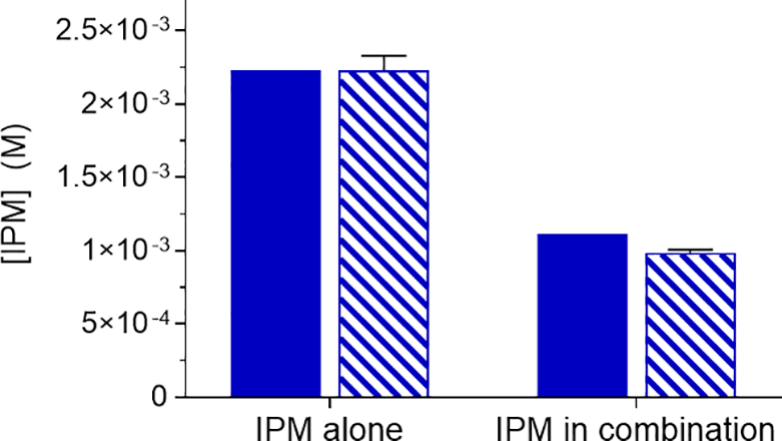
Predicted (solid bars) and experimental (striped bars) maximum achievable supersaturation of IPM in formulations containing IPM alone and in combination with FDN at a 1:1 M ratio. The values are an average of the triplicate measurements with error bars representing standard deviations.

Figure 9 displays the good agreement between the experimental and predicted concentrations of IPM using eq 4 for formulations containing either the drug alone or in combination with FDN at a 1:1 M ratio. FaSSIF had no pronounced effect on IPM solubilization similar to ATV. Therefore, eq 4 was applied and found to predict the concentration of IPM from formulations containing IPM and FDN at varied molar ratios (Supporting Information, Figure S5).

The maximum achievable concentration of FDN as a function of the amount of IPM added in the FaSSIF solution was found to be not significantly changing up to 1000 μg/mL of IPM added and to be significantly changing in the range of 2000 μg/mL and above (Supporting Information, Figure S6). This trend was also observed in our previous study on the solution chemistry of the FDN-IPM combination in buffer. The findings were explained by the complex nature of the solution and the colloidal phase evolving from this system and the properties of the drugs such as log P.^14^ It was neither possible to predict the behavior of FDN in buffer nor in FaSSIF using eq 4. However, eq 5 was found to predict the maximum achievable concentration of FDN at IPM concentrations below 1000 μg/mL and not at higher amounts of IPM added.

### Impact of FaSSIF and Multidrug Formulations on Drug Transport Across Membrane

Membrane transport through Caco-2 cells in buffer decreases in proportion to the content of drugs in a combination formulation.^12^ It is important to evaluate the membrane transport behavior of multidrug formulations in FaSSIF in order to correlate the findings with those in buffer, especially for the drugs that are highly solubilized by FaSSIF.

The interest in the supersaturated systems is due to their ability to increase the amount of drugs dissolved, and hence, their membrane transport. The mass transport of drugs is linearly related to the degree of supersaturation.^43^ The maximum achievable concentration and mass transport of a drug in solution occur at its amorphous solubility.^44^ Multiple studies on the impact of excipients and simulated intestinal media on solubilization have been conducted to understand the effect of improved solubility on membrane transport.^27,28^ These studies have shown that the mass transport–time profiles are the same for drugs in simple buffer and in complex solubilizing media, which clearly means that it is the degree of supersaturation—and not solubilization—which is a dominating factor in drug transport across a membrane.^43^ It is solubilization that increases the total concentration and not the thermodynamic activity of the drug in solution. However, it was shown that the partitioning of the excipients into the colloidal precipitate could decrease the membrane transport of the drug by decreasing its thermodynamic activity.^15^

This area is of great importance for understanding drug absorption in the gastrointestinal tract, but the literature is limited when it comes to amorphous multidrug formulations.^12^ Neither has the impact of complex solubilizing media on the membrane transport of drugs from multidrug formulations been widely explored.^27,28^

Our study therefore investigated the correlation of formulation dissolution and drug solubility with drug transport through an artificial membrane. The drugs in this study were affected differently by FaSSIF solubilization. RTV and FDN were solubilized by FaSSIF to a much greater extent than ATV and IPM (Table 2), which was also reflected in the dissolution profiles of the drugs (Figures 2 and 3). The solubility ratio, *R*_(Solubility)_, of the drugs in buffer and FaSSIF was compared to the flux ratio, *R*_(Flux)_, in the same media. The *R*_(Flux)_ ratio was very close to unity for all drugs, indicating that FaSSIF had no significant effect on their membrane transport (Supporting Information, Table S2). This was not the case for the *R*_(Solubility)_ ratio (Table 2) which indicated a clear solubility advantage for the FaSSIF medium. The solubilization advantage conferred by FaSSIF led to an overestimation of the true amount of drug transport across the membrane. A similar outcome is also reported for ATV and posaconazole in different solubilizing media.^27,28^ Clearly, the solubilization gained with FaSSIF diminishes upon the transport of the drug across the membrane.

The decrease in the maximum achievable concentration of drugs in combination during dissolution experiments in buffer is previously observed in an earlier study by our group.^14^ In contrast, the ATV-RTV system in the dissolution experiments performed in FaSSIF, showed different levels of the solubility decrease (Figure 2). The solubilization advantage of FaSSIF for RTV in combination was not observed in the membrane transport experiments. This was also the case when RTV was present alone, as no difference in mass transport–time profiles was observed in buffer and FaSSIF (Figure 4). In the case of the FDN-IPM system, FDN alone and in combination with IPM showed the same mass transport–time profiles in both media at the studied combination ratio. There was a slight increase in the flux of IPM when it is alone in FaSSIF, but the increase was not statistically significant from its flux in buffer, whereas the flux of IPM was significantly different in the case of the combination experiment with FDN (*p*-value < 0.05). The results may be attributed to different factors including the nature of the colloidal precipitate being a mixture of drugs with other properties than the ATV-RTV system: the latter two compounds are chemical analogues, whereas FDN and IPM are two different chemical classes commonly used in combination to treat hypertension. The exact mechanisms of the enhancement need to be further studied, but our results clearly show the risk of generalizing performance of multidrug formulations based on results from chemical analogues.

## Conclusions

The amorphous solubility of the drugs alone in FaSSIF was higher than that in buffer, but the improved solubility did not increase the membrane drug transport for any of the four drugs. For ATV and IPM from equimolar combination formulations, concentration and membrane transport were reduced roughly by 50% in both buffer and FaSSIF. In contrast, the concentration of RTV, from the equimolar formulation with ATV in FaSSIF, was only reduced 30% compared to the 50% in buffer. We demonstrated the solubility advantage gained by FaSSIF solubilization and established a model predicting the solution performance. However, the solubilization advantage did not translate into improved membrane transport; the membrane transport was reduced 50% in FaSSIF, similar to the reduction in buffer. The maximum achievable concentration of FDN in FaSSIF from the combination formulation with the IPM remained constant until the amount of IPM added was over 1000 μg/mL, where it started to decrease. On the other hand, the mass transport–time profile of FDN alone and in combination with IPM showed no significant difference in buffer and FaSSIF. The findings in this study are vital for the future development of multidrug formulations. They can be used to understand the impact of naturally available micelles and solubilizing systems and delineate the effects of formulation design on the resulting bioavailability.

## Supporting Information Available

Coefficient of determination (*R*^2^) of the equations obtained from the flux experiments; flux measurements of the model drugs in buffer and FaSSIF; calculations of the two solubilization barriers based on eqs 2 and 3; characterization of the crystalline form of ATV and RTV by PXRD and DSC; DSC heating curves of formulations containing 90% PVP and 10% drug(s); mass transport–time profiles at amorphous solubility of the model drugs: alone and in combination; concentration of FDN and IPM in the supernatant layer of ASDs containing both drugs at different molar ratios following their dissolution in FaSSIF; and concentration of FDN during a concentration titration of IPM in buffer and FaSSIF (PDF)
