# Supplementary material for: Impact of Simulated Intestinal Fluids on Dissolution, Solution Chemistry, and Membrane Transport of Amorphous Multidrug Formulations
Source: Mol Pharm. 2021 Oct 6;18(11):4079–89. doi: 10.1021/acs.molpharmaceut.1c00480 (PMC8564758; doi:10.1021/acs.molpharmaceut.1c00480)
Supplement: Supplementary file 1 — mp1c00480_si_001.pdf [file mp1c00480_si_001.pdf]

Impact of Simulated Intestinal Fluids on Dissolution, Solution Chemistry and Membrane Transport of Amorphous Multidrug Formulations

Mira El Sayed[1,2], Amjad Alhalaweh[2], and Christel A.S. Bergström[1]*

[1] Department of Pharmacy, Uppsala University, Biomedical Centre, P.O. Box 580, SE-751 23 Uppsala, Sweden.

[2] Recipharm OT Chemistry AB, SE-754 50 Uppsala, Sweden.

*Address correspondence to:

Prof. Christel Bergström
Department of Pharmacy
Uppsala University
P.O. Box 580
SE-751 23 Uppsala, Sweden
Email: christel.bergstrom@farmaci.uu.se
Phone: +46 – 18 471 4118
Fax: +46 – 18 471 4223

Table of Contents

Table S1. Coefficient of determination value, denoted by R^2, of the equations obtained from the flux experiments.

Table S2. Flux measurements of the model drugs in buffer and FaSSIF.

Table S3. Calculations of the two solubilization barriers based on eq 2 and eq 3.

Figure S1. Characterization of the crystalline form of ATV by PXRD and DSC. a) PXRD pattern and b) DSC heating curve.

Figure S2. Characterization of the crystalline form of RTV by PXRD and DSC. a) PXRD pattern and b) DSC heating curve.

Figure S3. DSC heating curves of formulations containing 90% PVP and 10% drug(s). ATV alone (green curve), ATV-RTV at 75:25 ratio (pink curve), ATV-RTV at 50:50 ratio (maroon curve), ATV-RTV at 25:75 ratio (blue curve) and RTV alone (red curve).

Figure S4. Mass transport-time profiles at amorphous solubility of the model drugs: alone a) ATV (in red) b) RTV (in yellow) c) FDN (in green) d) IPM (in blue) and in combination (in black). Circles (○) and triangles (△) represent buffer and FaSSIF, respectively. Error bars (4 replicates) show standard deviations.

Figure S5. Concentration of FDN (green) and IPM (blue) in the supernatant layer of ASDs containing both drugs at different molar ratios following their dissolution in FaSSIF. The lines represent the predicted concentrations based on eq 4 (dotted) or eq 5 (solid).

Figure S6. Concentration of FDN during a concentration titration of IPM in (△) buffer and (○) FaSSIF. The results of the buffer experiments were adapted from our previous work (reference 14 in the main manuscript).

Table S1. Regression coefficients, denoted by R^2, obtained from the linear curve fits from the flux experiments.

	Media	R^2 value			
		ATV	RTV	FDN	IPM
Drug alone	Buffer	0.9987	0.9972	0.9955	0.9996
	FaSSIF	0.9997	0.9991	0.9961	0.9998
Drugs in combination	Buffer	0.9989	0.9968	0.9984	0.9961
	FaSSIF	0.9986	0.9973	0.9923	0.9991

Table S2. Normalized flux values of the model drugs in buffer and FaSSIF.

Drug	Flux (mg/min/cm^2)		$R_{(flux)}$*
	Buffer	FaSSIF	
ATV	(5.06 ± 0.2) ×10^{-5}	(5.05 ± 0.1) ×10^{-5}	1.0
RTV	(2.46 ± 0.1) ×10^{-5}	(2.43 ± 0.1) ×10^{-5}	1.0
FDN	(1.15 ± 0.1) ×10^{-5}	(0.99 ± 0.2) ×10^{-5}	0.9
IPM	(1.87 ± 0.2) ×10^{-3}	(2.02 ± 0.1) ×10^{-3}	1.1

Results are the means ± standard deviations of four replicates.

$*R_{(\text{flux})} = \frac{\text{flux}_{(\text{FaSSIF})}}{\text{flux}_{(\text{buffer})}}$

Table S3. Calculations of the two solubilization barriers, $\log X_{(ideal)}$ and $\log \gamma$, based on equation 2 and 3.

drug	media	$\log X$	$\log X_{(ideal)}$	$\log \gamma$
ATV	buffer	-5.81	-3.19	2.62
	FaSSIF	-5.85	-3.19	2.66
RTV	buffer	-5.52	-1.80	3.72
	FaSSIF	-5.16	-1.80	3.36
FDN	buffer	-5.89	-1.26	4.63
	FaSSIF	-3.96	-1.26	2.70
IPM	buffer	-3.51	-1.59	1.91
	FaSSIF	-3.50	-1.59	1.90

log X: crystal lattice, log $X_{(ideal)}$: crystal lattice, log γ: activity coefficient

(a)

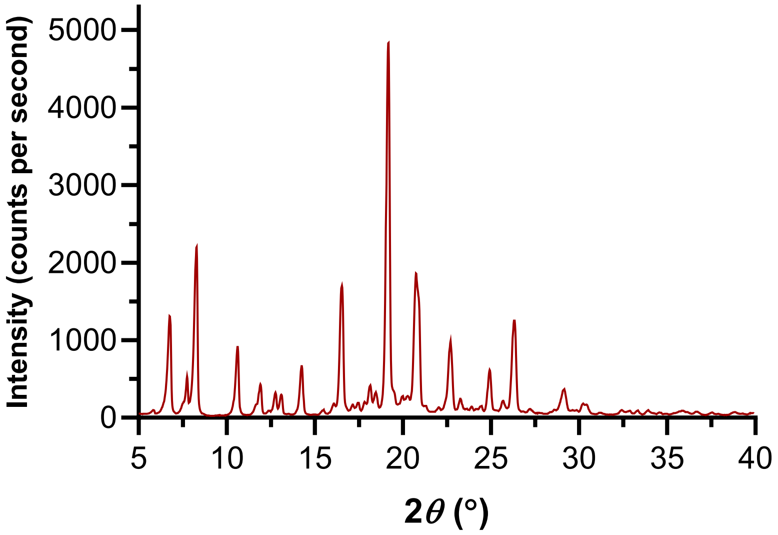

(b)

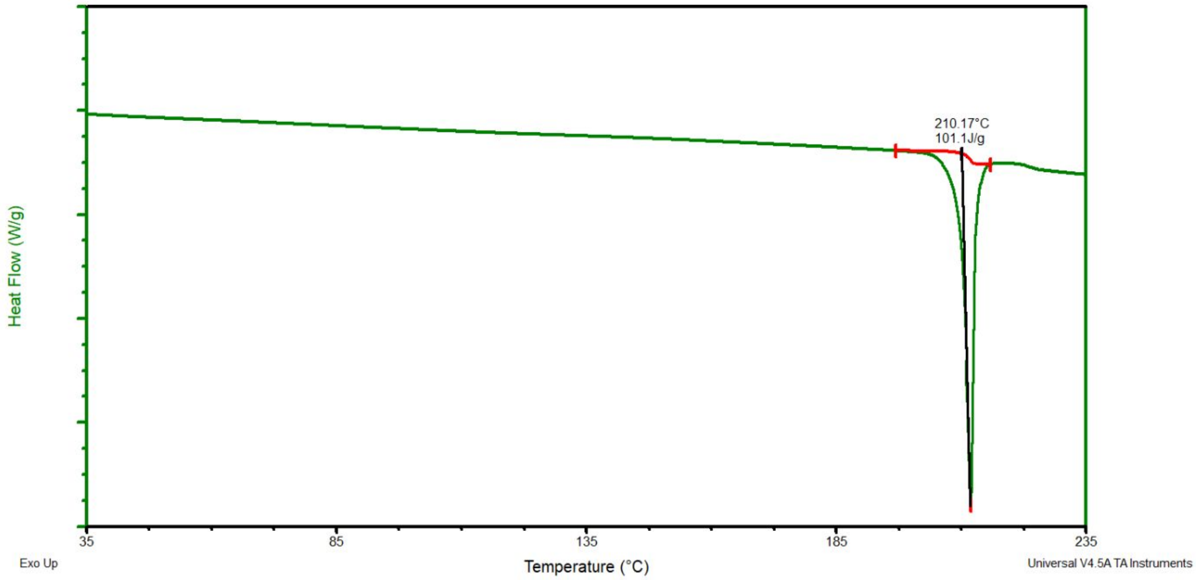

Figure S1. Characterization of the crystalline form of ATV by PXRD and DSC. a) PXRD pattern and b) DSC heating curve.

(a)

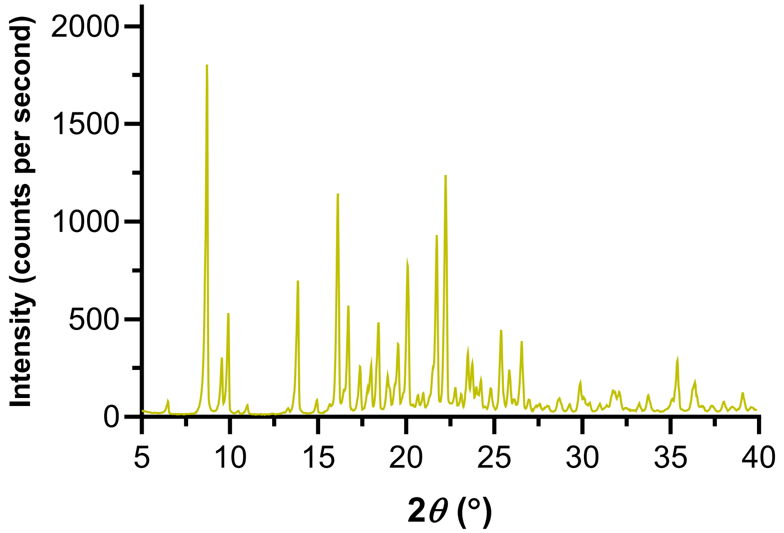

(b)

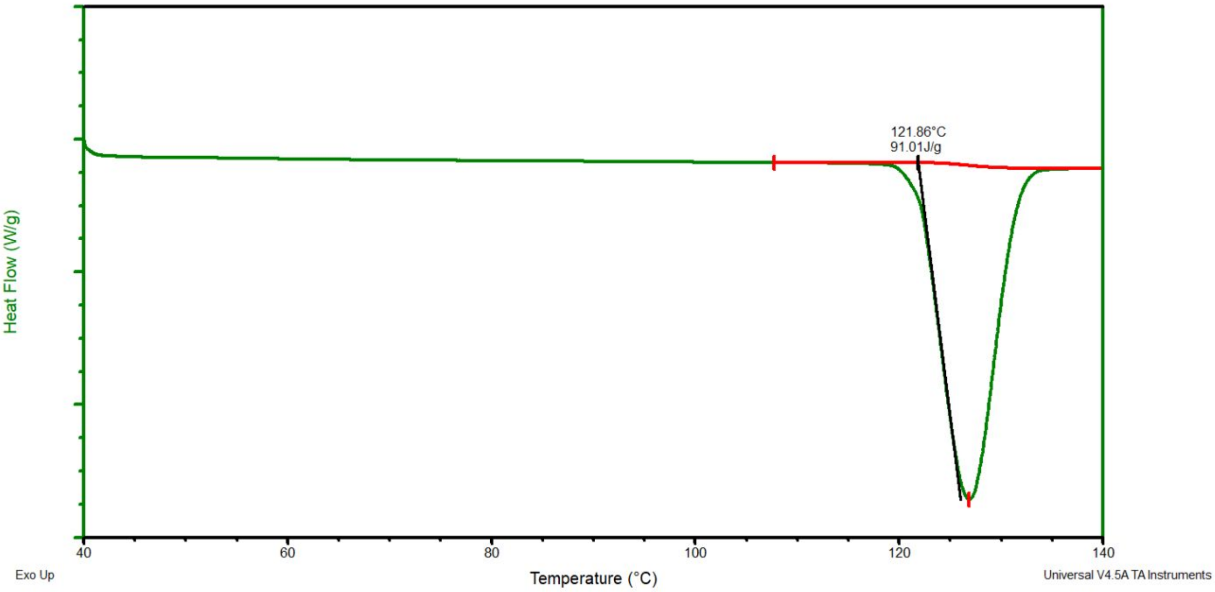

Figure S2. Characterization of the crystalline form of RTV by PXRD and DSC. a) PXRD pattern and b) DSC heating curve

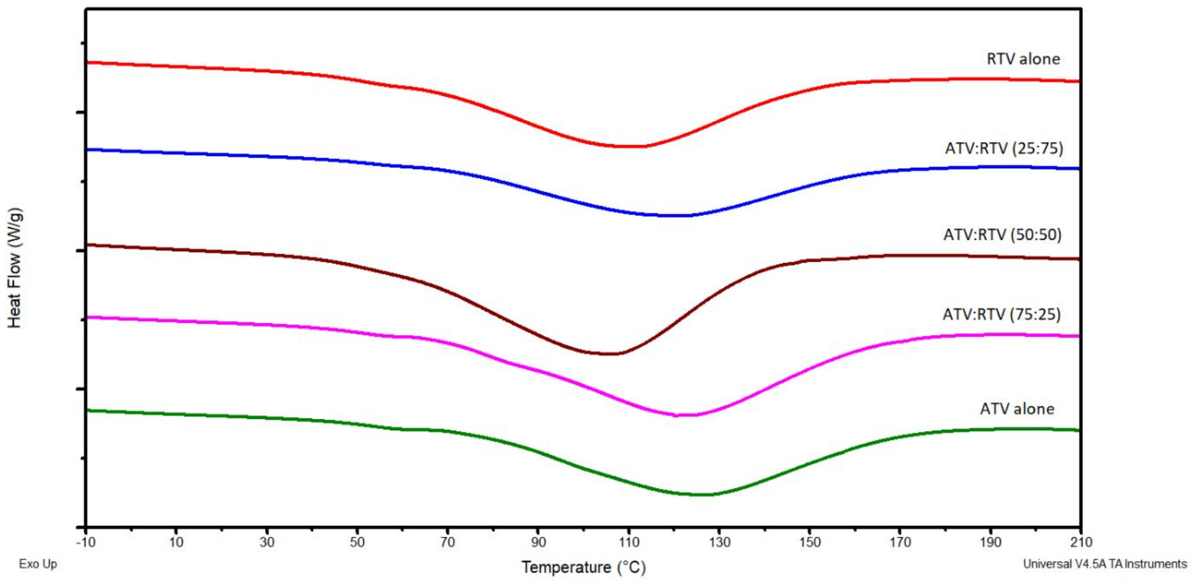

Figure S3. DSC heating curves of formulations containing 90% PVP and 10% drug(s). ATV alone (green curve), ATV-RTV at 75:25 ratio (pink curve), ATV-RTV at 50:50 ratio (maroon curve), ATV-RTV at 25:75 ratio (blue curve) and RTV alone (red curve).

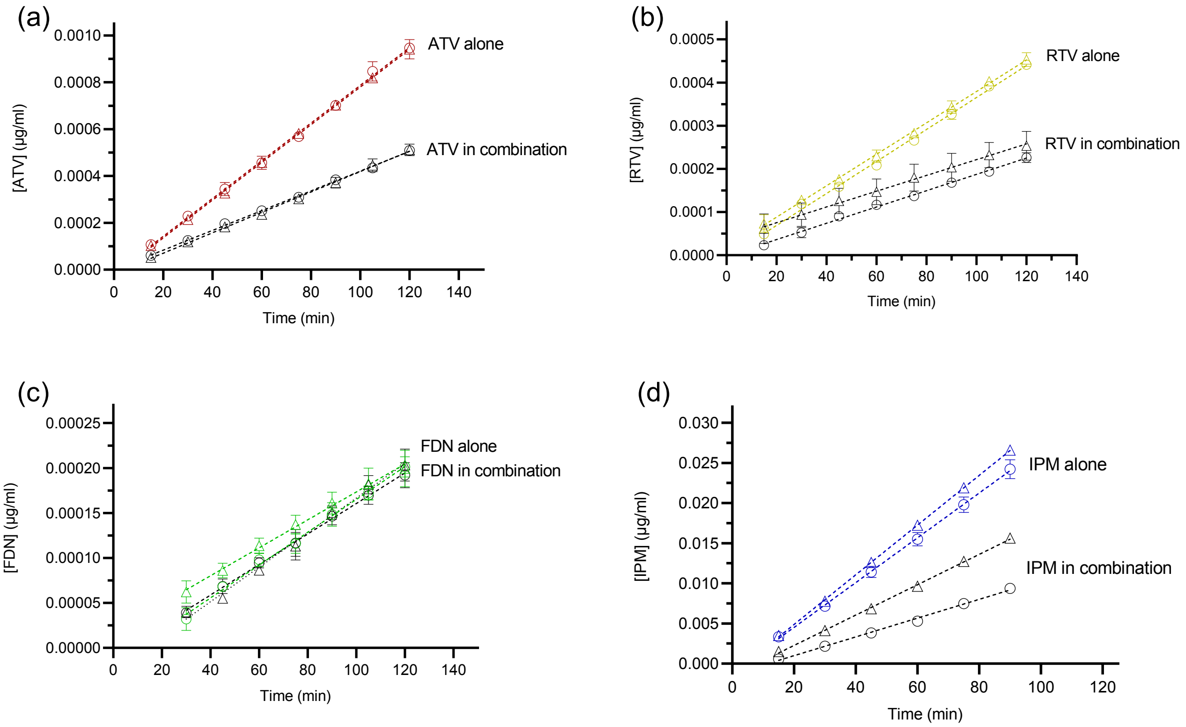

Figure S4. Mass transport-time profiles at amorphous solubility of the model drugs: alone a) ATV (in red) b) RTV (in yellow) c) FDN (in green) d) IPM (in blue) and in combination (in black). Circles (○) and triangles (△) represent buffer and FaSSIF, respectively. Error bars (4 replicates) show standard deviations.

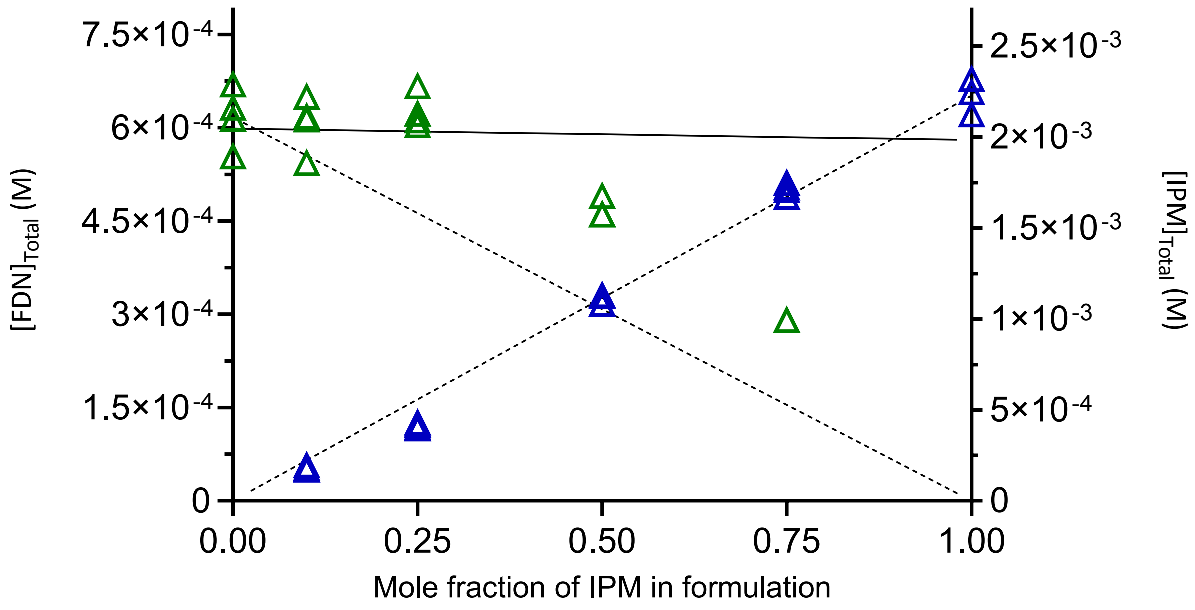

Figure S5. Concentration of FDN (green) and IPM (blue) in the supernatant layer of ASDs containing both drugs at different molar ratios following their dissolution in FaSSIF. The lines represent the predicted concentrations based on eq 4 (dotted) or eq 5 (solid).

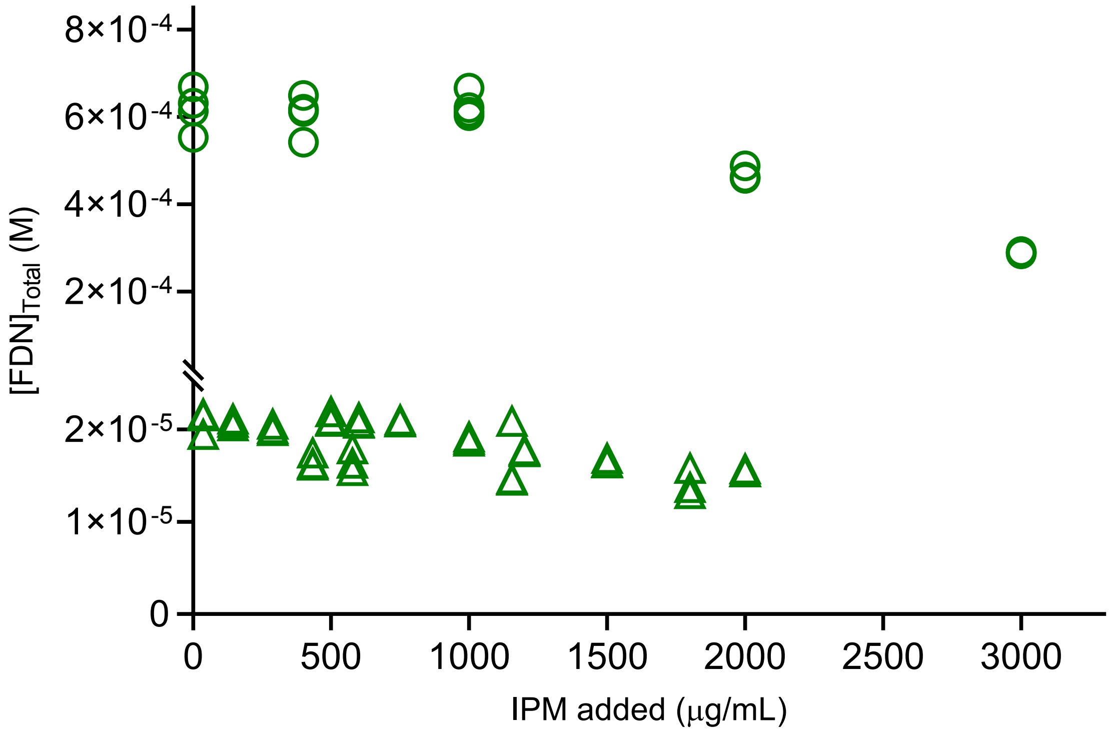

Figure S6. Concentration of FDN during a concentration titration of IPM in (△) buffer and (○) FaSSIF. The results of the buffer experiments were adapted from our previous work (reference 14 in the main manuscript).
